# American Dental Convention

**Published:** 1858-04

**Authors:** 


*American Dental Convention.*—We publish in another part of the present number of the Journal, some strictures on the proceedings of the last meeting of the American Dental Convention, over the signature of A. J. Volck, reflecting somewhat severely on the loose manner in which they were conducted; and, although we cannot endorse all the writer says, the objections urged by him against them, are not, it must be confessed, wholly without foundation. That the Convention has not hitherto been conducted in a manner altogether best calculated to secure the largest possible amount of practical and accurate scientific information, is, we think, undeniable. But it is scarcely to be expected, that an association composed of individuals taken indiscriminately from the ranks of comparatively a new profession—the portals of which are open to all, without regard to any fixed or recognized standard of qualification, should at once throw out transactions thoroughly systematized, and of a high scientific character. It has not, as yet, we believe, adopted a constitution for the government of its operations. Thus far, the workings of it have been mostly spontaneous and more or less desultory and irregular. It will, no doubt, however, ere long, become more thoroughly organized, and competent men, it is to be hoped, will then be appointed at each annual meeting to investigate carefully certain specified subjects, and report at the next succeeding convocation the result of their labors. When this shall be done, the meetings will be more interesting and instructive than at present.

If it has not hitherto elicited any elaborate scientific researches or voluminous reports, it has nevertheless been productive of good in bringing many members of the profession from opposite and far distant parts of the Union together, as it has afforded them an opportunity of becoming acquainted with each other, and of interchanging views with regard to their respective methods of practice. This has awakened a spirit of inquiry all over the land, which must, sooner or later, lead to important results.

Similar objections could have been urged against the American Society of Dental Surgeons seventeen years ago, although formed and conducted under a more perfectly organized system of rules, and yet, it contributed immensely to the advancement of practical dentistry. Still, no one, we think, will deny, that it did not commit many grave errors; and so has every association, which, for the most part, been composed of inexperienced men, not familiar with the usages of deliberative bodies and not possessed of high educational and scientific attainments. If, in the organization of societies for the advancement of dentistry, high professional qualifications were required as a test of membership, their transactions would certainly be more interesting and instructive, and as a consequence, productive of more good. We trust the day is not distant when such an association of dentists shall be formed. The time has now, it seems to us, surely arrived, when a sufficient number of scientific and liberal minded men in the profes-fession might be found to form such a society; and in the meantime, we trust the American Dental Convention will adopt a constitution and laws for the government of its proceedings.

We know it is easier to find fault with what others have done, than to do a thing in such a way as to be above criticism; and yet, criticism, when offered in a proper spirit, is of use, and no one ought to object to it.

